# Rare Bilateral Axillary Arch With Its Novel Insertion Into the Deltoid and Unilateral Supernumerary Head of the Sternocleidomastoid Muscle in a Single Cadaver

**DOI:** 10.7759/cureus.85096

**Published:** 2025-05-30

**Authors:** Hosne Ara, Adegbenro O Fakoya

**Affiliations:** 1 Cellular Biology and Anatomy, Louisiana State University (LSU) Health Shreveport, Shreveport, USA

**Keywords:** axillary arch muscle of langer, coracoid process, deltoid, latissimus dorsi, short head of biceps brachii, sternocleidomastoid muscle

## Abstract

Two rare and clinically significant anatomical variations were observed in a single cadaver during routine dissection at the Department of Cellular Biology and Anatomy of Louisiana State University (LSU) Health Shreveport in Shreveport, Louisiana. We report a rare case of a bilateral axillary arch muscle. On the right side, the muscle originated from the latissimus dorsi and merged with the short head of the biceps brachii, deltoid, and coracobrachialis muscles. On the left, it originated from the latissimus dorsi but was inserted into the fascia between the coracobrachialis and deltoid muscles. To our knowledge, this branching pattern finding has not been previously described in the literature. This anatomical variation may influence upper limb mobility and pose potential challenges during axillary surgical procedures and the diagnosis of compression syndromes. Additionally, in the same cadaver, an accessory head of the sternocleidomastoid muscle on the left side, originating from the middle third of the clavicle, was observed, which could impact neck mobility. Recognition of both variations holds clinical significance, contributing to advancements in anatomical knowledge relevant to surgery, radiology, and physical therapy.

## Introduction

The axillary arch muscle, also known as Langer's muscle or the axillopectoral muscle, is a rare muscular anomaly of the axilla. It is typically described as a slender muscle slip extending from the latissimus dorsi to the pectoralis major. So far, several researchers have documented different variations of this muscle anomaly. It has been observed that this muscle may occasionally attach to the coracoid process of the scapula, the medial epicondyle of the humerus, the teres major, the long head of the triceps brachii, the coracobrachialis, and the biceps brachii muscle [1]. This muscle's most usually described variation is when it extends from the latissimus dorsi to the coracoid process, the short head of the biceps brachii, or the pectoralis major muscle [1].

Regarding the distribution, the axillary arch can be unilateral or bilateral [2-4]. However, the embryological origin of this muscle has remained unclear [1]. In 1989, Wilson et al. proposed that the axillary arch muscle originates embryologically from the pectoralis major muscle mass, which explains its innervation by the medial pectoral nerve [5,6]. Subsequent reports have demonstrated that the thoracodorsal nerve innervates the muscle, likely due to its proximity to the latissimus dorsi muscle [1,2,6].

The sternocleidomastoid muscle is an important landmark during dissections of the triangles of the neck because it divides the neck into anterior and posterior triangles and is closely related to some important neurovascular structures. Typically, it consists of two heads: the sternal head and the clavicular head. The sternal head originates from the anterior surface of the manubrium of the sternum, and the clavicular head originates from the superior surface of the medial third of the clavicle. Both heads then ascend upward in parallel and eventually merge to form a single muscle belly, which inserts into the mastoid process of the temporal bone and onto the anterior portion of the superior nuchal line [5,6]. In 1963, Last reported four parts of the sternocleidomastoid muscle, which include the sternomastoid, sternooccipital, cleidomastoid, and cleidooccipital portions, which were again proved by several other researchers [7]. Some anatomists reported that these two heads can be completely separated from each other, which they considered normal [8]. Several groups reported that the sternocleidomastoid muscle comprised a superficial and a deep layer. The superficial layer consists of sternomastoid, sternooccipital, and cleidooccipital parts, and the deep layer consists of sternomastoid and cleidomastoid parts [8].

This case study presents rare variations of the neck and axillary regions, which could have potential implications for fields such as physical medicine, anesthesia, and gross and surgical anatomy.

## Case presentation

**Figure 1:**
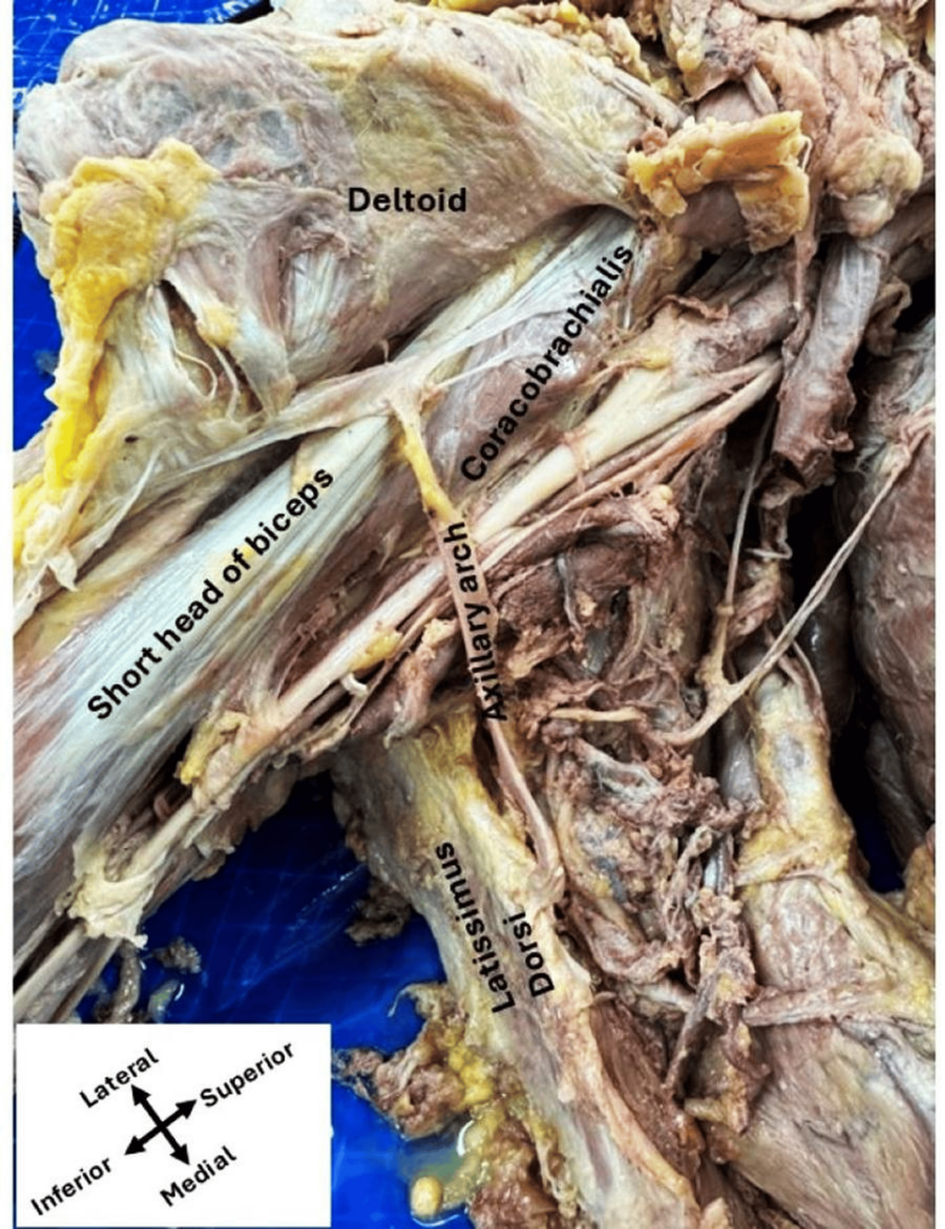
Right axillary dissection The axillary arch muscle originates from the anterior border of the latissimus dorsi, runs anterior to the axillary artery and brachial plexus branches, and inserts into the short head of the biceps brachii. Then it divides into two accessory slips in a "T" shape: the left slip merging with the fibers of the coracobrachialis muscle, while the right slip merges with the fibers of the deltoid muscle.

During the routine dissection of 24 cadavers for Allied Health students in the Department of Cellular Biology and Anatomy of Louisiana State University (LSU) Health Shreveport in Shreveport, Louisiana, we observed variations in the anterior neck and axillary region of cadavers. During the dissection of the axillary region, we identified a thin axillary arch muscle on the right side, originating from the anterior border of the latissimus dorsi muscle and inserted into the short head of the biceps brachii. This muscle then divides into two accessory slips in a "T" shape: the left slip merges with the fibers of the coracobrachialis muscle, while the right slip merges with the fibers of the deltoid muscle (Figure 1).

**Figure 2:**
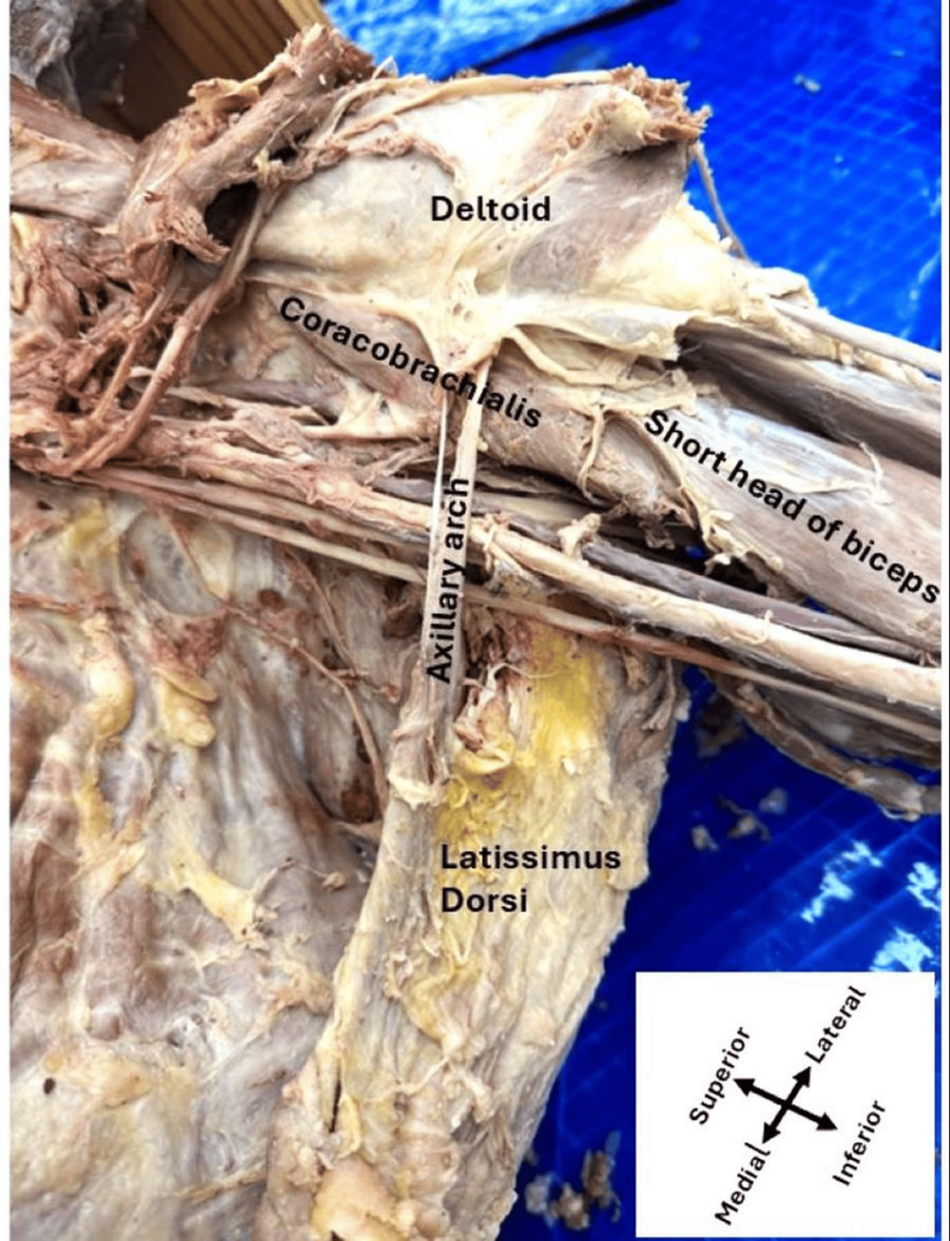
Photograph of the left axillary dissection showing the axillary arch muscle The axillary arch muscle originates from the latissimus dorsi, which runs anterior to the brachial plexus branches as a single thin slip and inserts directly into the fascia between the coracobrachialis and deltoid muscles.

On the left side, however, the axillary arch muscle also originated from the anterior border of the latissimus dorsi muscle. It was observed as a single (seemingly ripped), thin slip inserted directly into the fascia between the coracobrachialis and deltoid muscles without dividing into accessory slips (Figure 2). On both sides, the anterior axillary arch muscles course anterior to the axillary artery and brachial plexus branches, as shown in Figure 1 and Figure 2.

**Figure 3:**
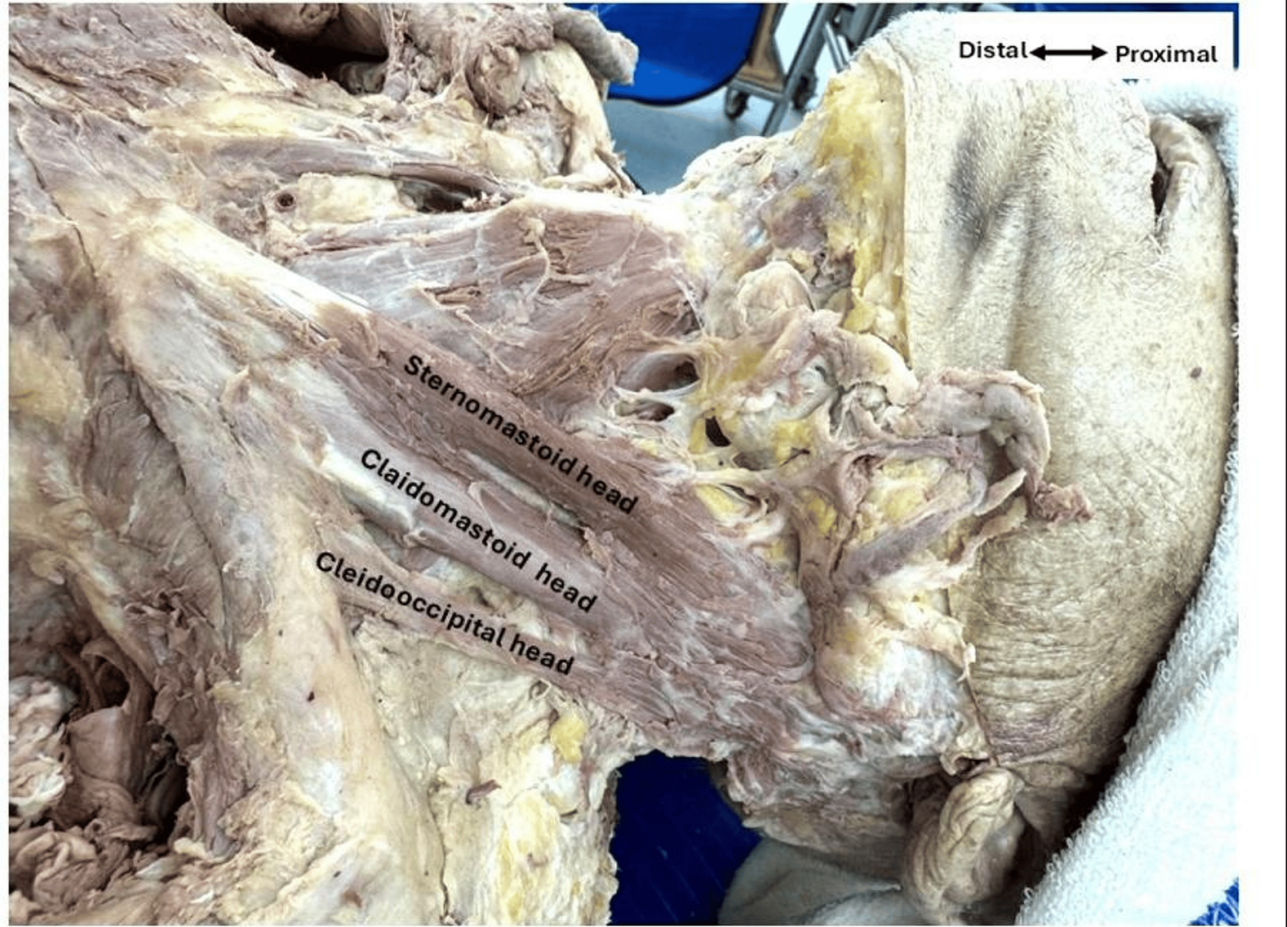
Left anomalous sternocleidomastoid muscle Left lateral view of the neck showing the sternomastoid, cleidomastoid, and cleidooccipital heads of the sternocleidomastoid muscle.

Additionally, during the dissection of the neck triangles, we observed a typical sternocleidomastoid on the right side, comprising the classical sternal head and clavicular head. However, on the left side, we identified three distinct heads of the sternocleidomastoid muscle. In our cadaver, we observed that the sternomastoid head originated from the superolateral portion of the sternum and inserted into the mastoid process, while the cleidomastoid head originated from the medial third of the clavicle, ascending independently to insert into the mastoid process without merging with the sternomastoid head. An accessory cleidooccipital head was identified, originating from the clavicle's middle third and inserted into the occipital bone as shown in Figure 3.

## Discussion

The axillary arch muscle was first described by Ramsay in 1795 [9]. Later, another group referred to the axillary arch muscle as the axillary arch of Langer or the axillopectoral muscle [1]. In 2007, Georgiev and colleagues showed that the most common variant of the axilla is the axillary arch muscle, which is still unknown to many expert surgeons. Their study showed that the frequency of the variations varies depending on the diverse groups of populations [10]. According to the previous report, the axillary arch originates from the latissimus dorsi but is inserted into multiple sites. The most common insertion sites of the axillary arch muscles are the pectoralis major, pectoralis minor, coracoid process, and biceps brachii muscle. In our current study, we recognized that on the right side, the axillary arch muscles originated from the latissimus dorsi and exhibited an extensive branching pattern with insertions into the coracobrachialis, the short head of the biceps brachii, and the deltoid muscle, which is a novel finding. However, it inserts solely into the fascia between the coracobrachialis and deltoid muscles on the left side.

Multiple anatomical studies have demonstrated that an axillary arch could complicate axillary surgeries, such as lymph node dissections or breast surgeries, by obscuring or compressing the underlying neurovascular structures, such as costoclavicular compression syndrome, axillary vein entrapment, and median nerve entrapment [11-13]. Therefore, to identify the etiology of compression syndrome in the cervico-axillary region, physicians should consider the possible presence of an axillary arch. Additionally, these axillary arch muscles may be clinically misinterpreted as soft tissue mass in imaging studies, which may lead to the requirement of unnecessary diagnostic procedures or incorrect diagnosis of the diseases.

In addition to the axillary arch muscle, we observed an accessory cleidooccipital head of the sternocleidomastoid muscle in the same cadaver. This rare anatomical variation could significantly impact neck movement, particularly in cases of trauma or during neck surgeries. This accessory cleidooccipital head during physical examination may lead to diagnostic confusion, as it could be mistaken for pathological tissue in imaging studies. Similarly, surgeons should know such variations to avoid unintentional injury during neck surgeries.

To our knowledge, this is the first report documenting an axillary arch muscle inserted into the deltoid and related fascia. However, since this is a cadaveric report, functional disturbances cannot be assessed, indicating that further studies are required to understand the functional impact of the axillary arch muscle.

Given the concurrent observation of these two rare muscular anomalies in the same cadaver, we aimed to explore a potential embryological correlation between the two findings. Embryologically, the axillary arch muscle, also known as Langer's arch, is regarded as a remnant of the panniculus carnosus. This subcutaneous muscle layer is prominent in lower mammals but largely regresses during normal human development [14,15].

The formation of the axillary arch muscle is linked to the migration and differentiation of myogenic precursor cells (myoblasts) derived from the hypaxial portion of the paraxial mesoderm, specifically from developing somites. These myoblasts populate the limb buds and thoracic wall to give rise to the muscles of the trunk and extremities [16]. Incomplete regression of the panniculus carnosus can lead to the persistence of accessory muscle slips, such as the axillary arch, representing vestigial muscular structures retained during development [15,17].

Interestingly, the axillary arch shares embryological features with other muscular anomalies, such as the three-headed variant of the sternocleidomastoid muscle. Though arising in different anatomical regions, both variations are hypothesized to result from common disturbances in embryonic myogenesis, particularly involving the segmentation, migration, or apoptosis of somite-derived muscle precursors [18,19]. These anomalies underscore the complex and dynamic processes involved in human muscle patterning and highlight the potential for phylogenetic remnants to persist into adulthood with clinical implications during surgical interventions in the axillary or cervical regions.

## Conclusions

These anatomical variations in the latissimus dorsi and sternocleidomastoid muscles highlight the importance of careful dissection and awareness among surgeons and clinicians. Recognizing these variations can deepen our knowledge of gross anatomy, help surgeons avoid complications during surgery, ensure accurate diagnoses in imaging, and elucidate the etiology of compression syndromes.
